# Epidemiology and clinical characteristics of infection/colonization due to carbapenemase-producing *Enterobacterales* in neonatal patients

**DOI:** 10.1186/s12866-022-02585-z

**Published:** 2022-07-12

**Authors:** Jiansheng Wang, Yuanpeng Lv, Weiwei Yang, Peng Zhao, Changfu Yin

**Affiliations:** 1 Clinical Laboratory, Hebei General Hospital, 348 Hepingxi Road, Shijiazhuang, 050051 Hebei China; 2 The Experimental Center, Clinic College of Hebei Medical University, 309 South Jianhua Street, Shijiazhuang, 050031 Hebei China

**Keywords:** Carbapenemase-producing, *Klebsiella pneumoniae*, *Escherichia coli*, Risk factors, Infection prevention

## Abstract

**Background:**

The aim of this study was to elucidate the epidemiological features of carbapenemase-producing *Enterobacterales* (CPE) in the pediatric and neonatal patients, to describe clinical characteristics of neonatal patients with CPE infections, and to assess risk factors for neonatal rectal colonization with CPE.

**Results:**

A total of 439 carbapenem-resistant *Enterobacterales* (CRE) isolates recovered from 367 infant patients were characterised, including 397 isolates of *Klebsiella pneumoniae* (KP) and 42 isolates of *Escherichia coli* (EC). Carbapenemase gene *blaNDM-1* was the most commonly detected, accounting for 86.56% (*n* = 380), followed by *blaKPC-2* (9.11%, 40) and *blaIMP-4* (4.33%, 19). MLST analysis showed 17 different STs detected within CPKP isolates, with ST20, ST2068, ST36 and ST17 being the most frequently isolated types. Eleven STs were identified within CPEC isolates, with ST325 being the dominant types. Eight isolates of NDM-1 producing KP, belonging to ST23, were identified as having hypervirulent traits. The main infections caused by CPE were pneumonia (*n* = 90) and sepsis (*n* = 16). All infected patients received monotherapy, with meropenem and ciprofloxacin being the most commonly used antibiotics. All pneumonia patients were cured or improved after treatment. Of the 16 patients with sepsis, 9 were cured or improved, 3 died, and 4 abandoned treatment without any clinical improvement. The rectal prevalences of CPE in the 0–3 days old (DO), the 4–28 DO, and the 29 DO-1 year old groups were decreased from 15.31%, 27.37% and 14.29% in the first stool screening period to 11.78%, 19.59% and 4.07% in the second stool screening period, respectively. Multivariate analysis showed that cesarean section, acidosis, respiration failure, gastric lavage and enema were independent risk factors for rectal colonization in the 0–3 DO group, whereas cesarean section, cephalosporins, gastric lavage and residence in rural area were independently associated with rectal colonization in the 4–28 DO group. The implementation of a series of evidence-based control measures eventually contained the CPE transmission.

**Conclusions:**

Continued vigilance, epidemiological studies, and multimodal infection prevention strategies are urgently needed due to frequent importations.

## Background

The spread of carbapenem-resistant *Enterobacterales* (CRE) into the pediatric population had become an extremely intractable problem due to their association with higher morbidity and mortality, longer hospital stays as well as limited therapeutic options [1–3]. Recent data from the CHINET surveillance system in China showed that imipenem-resistance rate of *Klebsiella pneumoniae* (KP) increased from 3.0% in 2005 to 25% in 2018 [4]. The annual upward trend was also obtained from the China Antimicrobial Resistance Surveillance System (http://www.carss.cn/), which showed that the annual isolation rate of carbapenem-resistant KP (CRKP) in 2014–2019 was 7.2/8.5/9.3/9.1/10.0/10.1 (%) for pediatric and newborn patients. The figure was unexpectedly high for neonatal patients, reaching 15.3%. However, very few detailed epidemiological data are available to uncover the underlying mechanisms for the increase.

*Enterobacterales*, especially *Klebsiella spp.* and *Escherichia coli* (EC), had become the most common pathogens in neonatal patients and were increasingly involved in nosocomial outbreaks in NICUs [5, 6]. CRE-associated outbreaks in neonatal patients in China had been increasingly reported [7–11]. Clonal spread and outbreaks of CRE in neonatal patients could bring severe consequences in terms of both morbidity and mortality [3, 12, 13]. Among these CRE isolates, NDM-1 producing KP had attracted much attention because KP with this resistant genotype had been considered to be the leading cause of neonatal carbapenem resistant sepsis in China [14]. Limited data existed on the treatment of CRE infections in neonatal patients. Available treatment regimens commonly used for adult CRE infections, such as polymyxins, aminoglycosides, tigecycline, and ceftazidime/avibactam, were restricted in the neonatal population due to poor safety and suboptimal pharmacokinetics [15]. Therefore, understanding the epidemiology and resistance patterns of infections in children was crucial for rational antimicrobial prescription and effective infection prevention [15].

Gastrointestinal CRE colonization in patients was an important risk factor for the dissemination of these bacteria in the healthcare settings and active rectal surveillance cultures to detect asymptomatic colonization in patients were very essential for the development of effective control measures [7, 16, 17]. Colonization also increased the likelihood of CRE infections [18, 19]. Prevention of transmission and determination of patients at highest risk were very essential for the control of CRE [1]. However, very few studies in China had investigated risk factors for colonization with carbapenemase-producing *Enterobacterales* (CPE) in neonatal patients.

During the period from April 2013 to April 2018, our neonatal ward had experienced an increasing number of CPE isolates reported by the clinical microbiology laboratory. The aim of the study was to determine their potential sources and the possible transmission modes in neonatal patients by performing a series of integrated analysis of clinical, epidemiologic, microbiologic and molecular data. We also described clinical characteristics of patients with CPE infection and evaluated risk factors for intestinal colonization with CPE in these patients.

## Results

### Genotypic characterization of neonatal and pediatric CPE isolates

A total of 160 CPE isolates were recovered from clinical samples of 153 infant patients under the age of 1 year admitted in both the neonatal and pediatric wards of our hospital during the period from April 2013 to April 2018. The sources of clinical microbiological cultures were bronchial aspirates or aspirating sputa (*n* = 135), blood samples (*n* = 16) and purulent fluids (*n* = 2). CPE prevalence especially occurred in newborns under three days, accounting for 72.5% (*n* = 116). Among the 160 CPE isolates, CPKP (96.88%, 155) was the most prevalent organism, including 96 (61.94%) NDM-1 producing isolates that belonged to 10 STs, 40 (25.81%) KPC-2 producing isolates of ST11 and 19 (12.26%) IMP-4 producing isolates of ST307. All 5 (3.13%) carbapenemase-producing EC isolates were NDM-1 producers that distributed into 3 STs (Table 1).

**Table 1:** Distribution of carbapenemase-producing *Enterobacterales* isolates in infant patients

	0–3 days age group					4–28 days age group					29–365 days age group			
	Sequence types of CPKP	No	Sequence types of CPEC	No		Sequence types of CPKP	No	Sequence types of CPEC	No		Sequence types of CPKP	No	Sequence types of CPEC	No
First stool screening	ST13 ST20 ST36 ST2068	2 49 18 41	ST1	1		ST13 ST20 ST36 ST2068 ST45	3 16 14 20 1	ST767 ST167 ST524	1 1 1		ST20 ST36 ST2068 ST2267	8 2 5 1	ST196 ST167 ST4542	1 2 1
Second stool screening	ST17 ST23 ST20	32 4 3	ST325	20		ST17 ST4 ST2068	12 4 1	ST325	6		ST17 ST23 ST45 ST685 ST36	1 1 1 1 2	ST398 ST469 ST83	1 1 1
Clinical specimens	ST11 ST13 ST20 ST36 ST39 ST307 ST2068 ST17 ST23 ST1786	29 1 33 13 13 15 4 2 2 1	ST471 ST469 ST325	1 1 1		ST11 ST20 ST36 ST2068 ST17 ST1655 ST307	8 9 2 2 3 1 3	ST325	1		ST11 ST20 ST39 ST36 ST719 ST23 ST307	3 4 1 3 1 1 1	ST325	1

*ST* sequence type, *CPKP* carbapenemase-producing *Klebsiella pneumoniae*, *CPEC* carbapenemase-producing *Escherichia coli*

A total of 1,650 patients (948 in the first stool screening period and 702 in the second stool screening period) were prospectively screened for CRE colonization during the two stool screening periods, 257 patients were identified to be colonized by carbapenem-resistant organisms (CROs) in which 244 cases were carried with NDM-1 producing *Enterobacterales* isolates. Among these colonized patients, 30 (12.3%) had developed pulmonary infections with NDM-1 producing KP of the same STs, which included ST36 (*n* = 11), ST20 (*n* = 11), ST2068 (*n* = 4), ST17 (*n* = 3) and ST23 (*n* = 1). Co-carriage of two NDM-1 isolates was found in 35 (14.34%) [21 (12.57%) of 167 in the first stool screening period vs 14 (18.18%) of 77 in the second stool screening period, *P* = 0.246] of the 244 patients. The rectal prevalence of CPE in the 29 days old (DO)-1 year old (YO) group was significantly decreased from 14.29% (15/105) in the first stool screening period to 4.07% (7/172) in the second stool screening period (*P* = 0.002). The rectal prevalences of CPE in both the 0–3 DO and the 4–28 DO groups in the second stool screening period were decreased, but no statistical significances were reached (15.31%, 100/653 vs 11.78%, 51/433, *P* = 0.099 and 27.37%, 52/190 vs 19.59%, 19/97, *P* = 0.148, respectively).

In the first stool screening period, 188 CPE strains were isolated, including 180 NDM-1 KP of 6 STs, with ST20 (40.56%, 73), ST2068 (36.67%, 66), and ST36 (18.89%, 34) being the 1st, 2nd and 3rd most common types, and 8 NDM-1 EC of 6 STs (Table 1). Although transmission events continued to occur during the second stool screening period, isolates of some ST types were different from those in the first stool screening period, and all 91 isolates were NDM-1-producers consisting of 62 KP of 8 STs with a predominance of ST17 (72.58%, 45) and 29 EC of 4 STs with ST325 (89.66%, 26) being the major type (Table 1).

All CPE isolates were resistant to cephalosporins, β-lactams and β-lactamase inhibitor combinations and carbapenems. KPC-2 KP isolates also exhibited resistance to aminoglycosides and fluoroquinolones, while metallo-β-lactamase (IMP-4 or NDM-1)-producing strains were generally susceptible to aminoglycosides and fluoroquinolones.

### Clinical characteristics of neonatal CPE infection

During the study period, a total of 115 patients aged less than 3 days had nosocomial CPE acquisitions, 25 of whom had respiratory tract colonization. The remaining patients (*n* = 90) developed infections caused by CPE, in which pneumonia and sepsis accounted for 84.44% (76/90) and 15.56% (14/90), respectively.

The demographic and clinical characteristics of the 76 patients with pneumonia were summarized in Table 2. The great majority of patients were males (72.37%, 55/76), 80.26% (61/76) were born prematurely, and 67.11% (51/76) were born by cesarean delivery. The main comorbid conditions were anemia (56.58%), hypocalcemia (52.63%), hypoxic-ischemic encephalopathy (48.68%), pulmonary membrane disease (46.05%), and respiratory failure (42.11%). All but two patients were given gastric lavage, 72.37% (55/76) had received mechanical ventilation, and 31.58% (24/76) had peripherally inserted central catheters placed. All 76 infected patients received antimicrobial treatment before CPE isolation, with β-lactam/β-lactamase inhibitor combinations (77.63%) being the most frequently used antibiotics, followed by cephalosporins (31.58%) and carbapenems (23.68%). Among the CPE groups with different carbapenemase types, the most commonly used antibiotics varied prior to CPE isolation, with β-lactam/β-lactamase inhibitor combinations (100%) in the IMP-4 and KPC-2 groups, and β-lactam/β-lactamase inhibitor combinations (58.54%) and cephalosporins (56.10%) in the NDM-1 group. After the culture results were obtained, 81.58% (62/76) changed antimicrobial therapy. Meropenem was the most frequently administered antibiotic, especially in the KPC-2 group (100%) and NDM-1 group (79.41%), followed by ciprofloxacin in the IMP-4 group (58.33%) and NDM-1 group (17.65%). Of the 76 patients treated with antibiotics, all were cured or improved regardless of whether the antibiotic regimen was changed.

**Table 2:** Demographic and clinical characteristics of seventy-six patients with CPE pneumonia according to carbapenemase types

Variables	Total (*n* = 76) No. (%)	IMP-4 (*n* = 13) No. (%)	KPC-2 (*n* = 22) No. (%)	NDM-1 (*n* = 41) No. (%)
Gender (male)	55 (72.37)	9 (69.23)	16 (72.73)	30 (73.17)
Prematurity	61 (80.26)	11 (84.62)	18 (81.82)	32 (78.05)
Cesarean section	51 (67.11)	8 (61.54)	17 (77.27)	26 (63.41)
Gestational age (d)	237.09 ± 23.34	233.08 ± 17.62	231.55 ± 23.35	241.34 ± 26.27
Birth weight (g)	2103.62 ± 876.30	2003.08 ± 752.85	1857.95 ± 728.66	2267.32 ± 962.85
Hospital stay (d)	24.82 ± 13.22	24.54 ± 10.18	27.32 ± 14.27	23.59 ± 13.59
Hospital stay before CPE isolation (d)	7.64 ± 5.72	9.69 ± 6.05	7.91 ± 4.97	6.85 ± 5.95
Hypocalcemia	40 (52.63)	2 (15.38)	12 (54.55)	26 (63.41)
Respiratory distress syndrome	29 (38.16)	4 (30.77)	7 (31.82)	18 (43.90)
Acidosis	27 (35.53)	3 (23.08)	9 (40.91)	15 (36.59)
Anemia	43 (56.58)	7 (53.85)	14 (63.64)	22 (53.66)
Hypoxic-ischemic encephalopathy	37 (48.68)	9 (69.23)	10 (45.45)	18 (43.90)
Acleistocardia	29 (38.16)	5 (38.46)	1 (4.55)	23 (56.10)
Pulmonary hypertension	11 (14.47)	1 (7.69)	0 (0)	10 (24.39)
Respiratory failure	32 (42.11)	6 (46.15)	6 (27.27)	20 (48.78)
Pulmonary membrane disease	35 (46.05)	7 (53.85)	14 (63.64)	14 (34.15)
Nasogastric feeding	55 (72.37)	8 (61.54)	17 (77.27)	30 (73.17)
Gastric lavage	74 (97.37)	11 (84.62)	22 (100)	41 (100)
Enema	24 (31.58)	0 (0)	7 (31.82)	17 (41.46)
Mechanical ventilation	55 (72.37)	9 (69.23)	14 (63.64)	32 (78.05)
Peripherally inserted central catheter	24 (31.58)	4 (30.77)	8 (36.36)	12 (29.27)
Infection with *Klebsiella pneumoniae*	74 (97.37)	13 (100)	22 (100)	39 (95.12)
Infection with *Escherichia coli*	2 ( 2.63)	0 (0)	0 (0)	2 (4.88)
Previous antibiotic exposure				
β-lactam/β-lactamase inhibitor combinations	59 (77.63)	13 (100)	22 (100)	24 (58.54)
Gentamicin	5 (6.58)	3 (23.08)	0 (0)	2 (4.88)
Cephalosporins	24 (31.58)	0 (0)	1 (4.55)	23 (56.10)
Carbapenems	18 (23.68)	5 (38.46)	5 (22.73)	8 (19.51)
Change in antibiotic treatment after CRE isolation	62 (81.58)	12 (92.31)	16 (72.73)	34 (82.93)
Ciprofloxacin	13 (17.11)	7 (58.33)	0 (0)	6 (17.65)
Meropenem	47 (61.84)	4 (33.33)	16 (100)	27 (79.41)
β-lactam/β-lactamase inhibitor combinations	2 (2.63)	1 (8.33)	0 (0)	1 (2.94)
Outcome				
Cure	63 (82.89)	12 (92.31)	17 (77.27)	34 (82.93)
Improvement	13 (17.11)	1 (7.69)	5 (22.73)	7 (17.07)

*CPE* carbapenemase-producing *Enterobacterales*

Fourteen patients aged less than 3 days, 7 males and 7 females, developed sepsis, including 2 cases with IMP-4 KP, 5 cases with KPC-2 KP and 7 cases with NDM-1 KP. Among these patients, 8 were premature and 10 were born via a cesarean deliveries. The mean gestational age was 234.57 ± 17.51 days and the mean birth weight was 1890.00 ± 678.75 g. The mean length of hospital stay was 23.43 ± 17.52 days. Twelve patients were given gastric lavage, 7 received nasogastric tube feeding, 9 had been subjected to mechanical ventilation, and 5 had peripherally inserted central catheters placed. All the 14 patients received β-lactam/β-lactamase inhibitor combinations treatment before CPKP isolation, two of whom also received cephalosporins treatment. Antimicrobial therapy was changed in 12 patients after culture results were available. Among them, one NDM-1 KP infected patient was treated with ciprofloxacin and cured, 11 were treated with meropenem, 8 of them were cured or improved, 2 died from IMP-4 KP infections, and 1 died from NDM-1 KP infection. The remaining two KPC-2 KP infected patients who continued to receive β-lactam/β-lactamase inhibitor combinations abandoned treatment without any clinical improvement.

In the 4–28 DO group, 16 of 25 patients contracted infections caused by CPKP, including 2 KPC-2 KP sepsis, 6 KPC-2 KP pneumonia, 7 NDM-1 KP pneumonia and 1 IMP-4 KP pneumonia. The infected patients were 11 males and 5 females, with a mean age of 17.69 ± 7.21 days. The mean gestational age of the patients was 250.38 ± 22.66 days, and the mean birth weight was 2387.81 ± 875.04 g. The mean length of hospital stay was 14.88 ± 5.34 days. Three ptients were preterm and six were cesarean deliveries. Ten patients received nasogastric tube feeding and 2 received mechanical ventilation. None of the patients received peripherally inserted central catheters. After CPKP isolation, 11 patients were treated with meropenem, 3 treated with ciprofloxacin, and 2 treated with β-lactam/β-lactamase inhibitor combinations. Following antibiotic treatment, all pneumonia patients, including 5 community-acquired infections and 9 hospital-acquired infections, were cured or improved, while the two nosocomial sepsis patients (one male and one female) abandoned treatment without clinical improvement after meropenem therapy.

### Risk factors for rectal colonization with CPE in neonatal patients

Among the 1,650 patients with rectal screening cultures, 1373 were neonatal patients, of whom 696 had sterile cultures and 677 had positive cultures, including 285 with carbapenem-susceptible gram-negative bacilli (CSGNB), 157 with gram-positive cocci, and 235 with CROs. Of the 235 CRO patients, 222 cases colonized by NDM-1 producing *Enterobacterales* were included in the case group. The case-matched control group was 194 patients with carbapenem-susceptible *Enterobacterales* (CSE) selected from 285 CSGNB patients. In the univariate analysis, cesarean section, acleistocardia, cephalosporins, gastric lavage, nasogastric feeding and residence in rural area were identified as risk factors for colonization with CPE in the 4–28 DO group, while gestational age, birth weight, prematurity, cesarean section, respiratory distress syndrome, acidosis, hyperbilirubinemia, respiration failure, hypocalcemia, meropenem, oxygen inhalation, sputum suction, gastric lavage, nasogastric feeding, enema and endotracheal intubation were significantly associated with CPE colonization in the 0–3 DO group (Table 3). In multivariable logistic analysis, only cesarean section, cephalosporins, gastric lavage and residence in rural area remained as risk factors independently associated with CPE colonization in the 4–28 DO group, whereas cesarean section, acidosis, respiration failure, gastric lavage and enema were independent risk factors for CPE colonization in the 0–3 DO group (Table 3).

**Table 3:** Risk factors assoclated with rectal colonization of NDM-1-producing *Enterobacterales* in neonatal patient

Variables	4-28 days age group					0-3 days age group				
	Carbapenem-susceptible *n* = 44 (%)	Carbapenem-resistant *n* = 71 (%)	Univariate analysis *P* value	Multivariate analysis OR (95% CI) *P* value		Carbapenem-susceptible *n* = 150 (%)	Carbapenem-resistant *n* = 151 (%)	Univariate analysis *P* value	Multivariate analysis OR (95% CI) *P* value	
Age of admission (d)	9.47 ± 7.48	10.18 ± 7.27	0.222							
Gender (male)	90 (62.5)	40 (56.34)	0.385			78 (52)	80 (52.98)	0.913		
Gestational age (d)	271.67 ± 25.26	270.30 ± 14.39	0.069			267.77 ± 18.65	250.57 ± 23.81	< 0.001		
Birth weight (g)	3306.33 ± 568.21	3239.65 ± 526.20	0.592			3080.97 ± 665.74	2637.55 ± 880.633	< 0.001		
Prematurity	10 (6.94)	8 (11.27)	0.282			42 (28)	93 (61.59)	< 0.001		
Cesarean section	54 (37.5)	43 (60.56)	0.001	2.55 (1.37–4.74)	0.003	66 (44)	100 (66.23)	< 0.001	2.92 (1.69–5.05)	< 0.001
Pulmonary infection	34 (23.61)	20 (28.17)	0.469			18 (12)	23 (15.23)	0.414		
Respiratory distress syndrome	12 (8.33)	5 (7.04)	0.741			96 (64)	122 (80.79)	0.001		
Acidosis	1 (0.69)	1 (1.41)	0.552			22 (14.67)	53 (35.10)	< 0.001	2.25 (1.17–4.33)	0.016
Hyperbilirubinemia	108 (75)	47 (66.20)	0.176			45 (30)	13 (8.61)	< 0.001		
Respiration failure	1 (0.69)	2 (2.82)	0.254			7 (4.67)	38 (25.17)	< 0.001	3.46 (1.41–8.45)	0.007
Acleistocardia	67 (46.53)	44 (61.97)	0.033			96 (64)	83 (54.97)	0.139		
Hypocalcemia	6 (4.17)	3 (4.23)	> 0.999			31 (20.67)	59 (39.07)	< 0.001		
Anemia	15 (10.42)	10 (14.08)	0.430			11 (7.33)	18 (11.92)	0.177		
β-lactam/β-lactamase inhibitor	34 (23.61)	20 (28.17)	0.469			46 (30.67)	52 (34.44)	0.485		
Cephalosporins	52 (36.11)	40 (56.34)	0.005	2.12 (1.14–3.94)	0.017	79 (52.67)	94 (62.25)	0.093		
Meropenem	10 (6.94)	6 (8.45)	0.692			2 (1.33)	10 (6.62)	0.019		
Oxygen inhalation	26 (18·06)	15 (21.13)	0.590			60 (40)	100 (66.23)	< 0.001		
Sputum suction	62 (43.06)	36 (50.70)	0.290			108 (72)	143 (94.7)	< 0.001		
Gastric lavage	11 (7.64)	16 (22.54)	0.002	3.44 (1.44–8.22)	0.005	97 (64.67)	138 (91.39)	< 0.001	3.09 (1.49–6.39)	0.002
Nasogastric feeding	12 (8.33)	13 (18.31)	0.032			56 (37.33)	85 (59.29)	0.001		
Enema	16 (11.11)	13 (18.31)	0.146			40 (26.67)	91 (60.26)	< 0.001	2.84 (1.65–4.91)	< 0.001
Endotracheal intubation						7 (4.67)	32 (21.19)	< 0.001		
Residence in rural area	46 (31.94)	37 (52.11)	0.004	2.22 (1.19–4.13)	0.012	72 (48)	72 (47·68)	0.956		

*Abbreviations: OR* odds ratio, *CI* confidence interval

### Epidemiological change of CPE in neonatal patients

In April 2013, a sporadic epidemic of NDM-1 KP ST39 clone occurred in neonatal ward, involving 13 cases less than 3 days old, all of whom were nosocomially acquired. This clone was initially isolated in March 2013 from a 5-month-old child with community-acquired pneumonia in the pediatric ward and might subsequently be transmitted to the neonatal ward via rotating medical staff. But it disappeared in December 2013. In January and July 2014, IMP-4 KP ST307 and KPC-2 KP ST11 clones were separately introduced into the ward and then spread rapidly, affecting 18 and 37 clinical cases, respectively. Between November and December 2014, NDM-1 KP ST20 was detected in three neonatal patients. Despite the implementation of basic infection control practices, especially reinforced hand hygiene before and after patient contact, the spread of these clones could not be controlled. Routine environmental screening showed that CPKP could be isolated from the ward environment and the hands of medical staff, including 2 isolates of CPKP ST39 and CPKP ST20 from rubber ring of incubators, 2 isolates of CPKP ST39 and CPKP ST307 from humidified water, 2 isolates of CPKP ST20 and CPKP ST11 from incubator handles, 1 isolate of CPKP ST20 from object surface and 1 isolate of CPKP ST20 from medical staff hands. This suggested an important model of transmission via the hands of healthcare workers from the contaminated environment to new patients. Considering that the neonatal ward and ICU were located on the same floor, and that adult ICU at that time had experienced the epidemic of KPC-2 KP ST11 clones, it was speculated that there existed an important route of ‘non-patient transfer’ transmission between the two wards. So a new ward was opened in another building at the end of 2014 to receive new admissions. Those who had been affected remained in the original ward until they were discharged. Extensive environmental screening cultures were performed one month before and after admission to the new ward, but did not yield any CRE.

**Fig. 1:**
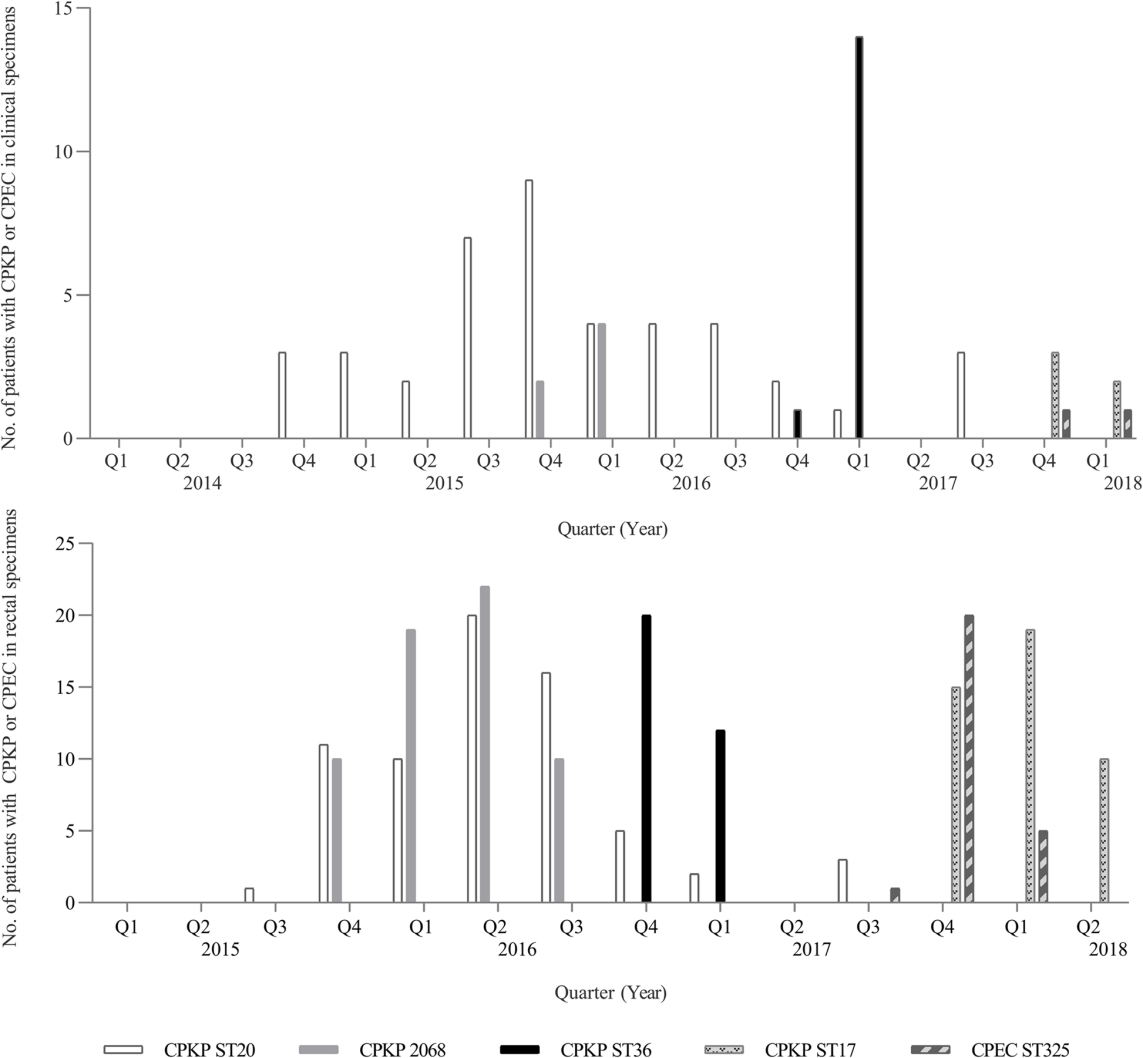
Distribution of neonatal patients with NDM-1 producing *Klebdieella pneumoniae* or *Escherichia coli* of common STs according to the quarters. Abbreviations: ST, sequence type; CPKP, carbapenemase-producing *Klebdieella pneumoniae*; CPEC, carbapenemase-producing *Escherichia coli*

In January 2015, NDM-1 KP ST20 clone re-emerged and spread in the new ward. To determine the probable sources of the pandemic strains, active rectal screening cultures were initiated for infant patients under one year old. To confirm the existence of the maternal–neonatal transmission of CRE, 342 stool or perianal samples and 286 amniotic fluid samples collected from 342 pregnant women with suspected intrauterine infections in the maternity ward were screened for CRE, but no isolates were identified to be carbapenem-resistant. Therefore, the likely route of CPE through vertical transmission from the mothers at birth was excluded. The fact that there were no CPE circulating in the maternity ward and that no CPE had been isolated from routine obstetric environmental screenings did not support the transmission from the maternity to the neonatal ward. In November 2015, the new ward was partially closed to external admissions, as the transmission of NDM-1 KP ST20 and KP ST2068 clones could not be inhibited (Fig. 1).

In April 2016, the initial results of rectal cultures were fed back to the ward, pointing out that the rectal colonization of NDM-1 strains in neonatal patients and their subsequent transmissions should be considered as key elements for intervention. Immediately, the frequency and extent of environmental disinfection with chlorine-based compound were increased and enhanced, especially the carriers’ surroundings, and educational or training programs on hand hygiene were regularly provided, particularly for rotating medical staff, due to possible transmission between the pediatric and neonatal wards. Afterward, the number of new cases gradually decreased (Fig. 1). Multiple environmental samples showed no detectable CROs in the new ward environment. In October 2016, a new NDM-1 KP ST36 clone emerged and spread (Fig. 1). The multi-disciplinary team identified limited space and understaffing as important contributory factors in the failure to control the spread of CRKP. This prompted the institution and implementation of additional measures, including the reduction of the total number of beds in the room from 14 to 10 and the increase in the number of medical staff. The implementation of these measures resulted in a significant reduction in the number of new cases.

**Fig. 2:**
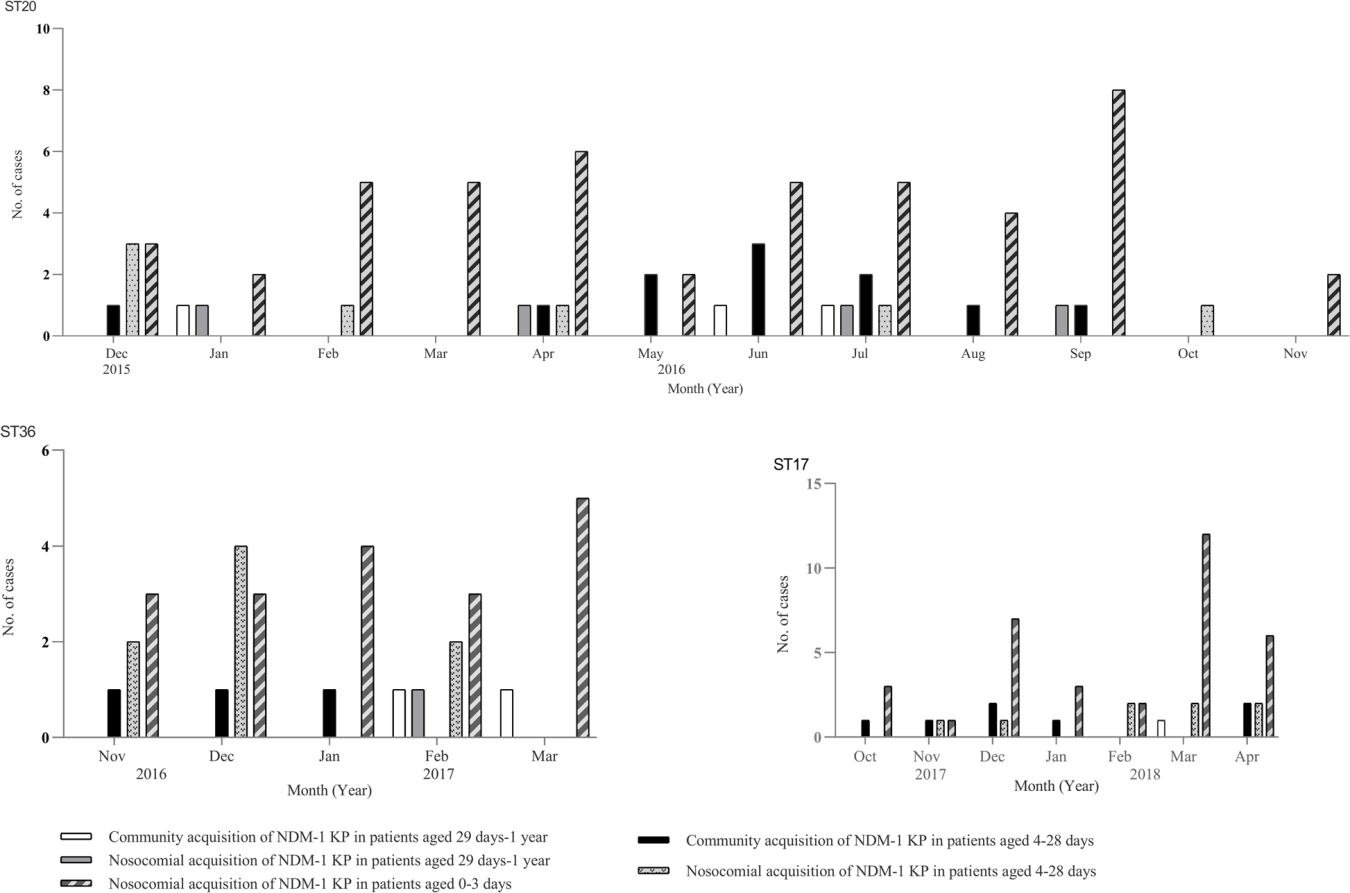
Distribution of infant patients with NDM-1 producing *Klebdieella pneumoniae* strains of three representative STs according to months. Abbreviations: KP, *Klebdieella pneumoniae*; ST, sequence type

In September and October 2017, two new NDM-1 strains of EC ST325 and KP ST17 were separately introduced and silently spread (Fig. 1), suggesting that these measures were far from sufficient to avoid silent transmission. We then separately performed prospective matched case–control studies to determine neonatal risk factors associated with rectal colonization of CPE in the 0–3 DO and the 4–28 DO groups. In the multivariate analysis, we found that gastric lavage and enema were independent risk factors for colonization in the 0–3 DO group, whereas gastric lavage and cephalosporins were risk factors independently associated with colonization in the 4–28 DO group. These results strongly suggested that improperly medical procedures and irrational use of antibiotics had probably played relatively direct causal roles in neonatal CPE colonization and transmission. In addition, a detailed analysis of patient admission data combined with cultures and molecular typing showed that multiple or repeated independent introductions of diverse NDM-1 strains into the neonatal ward direct from the community through colonized/infected infant patients, especially patients older than 3 days, resulted in continued clonal transmission events (Fig. 2). So strict aseptic procedures were emphasized and monitored to insure stringent compliance in caring for high-risk patients, active stool surveillance cultures upon admission were listed as a routine requirement, and antibiotic stewardship efforts were also enhanced to limit the unnecessary use of antibiotics in this population. With these enhanced interventions, sporadic new CPE cases were still identified, but no outbreaks occurred.

### Epidemiological features of hypervirulent CPKP

Nine virulence genes were tested in all carbapenemase-producing KP isolates, and 8 NDM-1 isolates were positive, all of which belonged to ST23. In the neonatal ward, a total of 5 neonatal patients aged less than 3 days old were found to be infected or colonized with CPKP ST23 between November 2017 and April 2018, including one case with pulmonary infection and four cases with rectal colonization. The five affected patients were nosocomially acquired and dispersedly distributed. Among the four neonatal patients with rectal colonization, one developed pulmonary infection caused by the same CPKP ST23. CPKP ST23 was also isolated from pus of a 1-month-old infant patient and stool sample of a 2-month-old infant patient in pediatric ward in February and March 2018, respectively. After the implementation of the above multiple intensified control measures, this strain had not been detected since May 2018.

## Discussion

In the study, we detected a high prevalence of rectal colonization with NDM-1 producing *Enterobacterales* among neonatal patients due to nosocomial transmission. We also found that multiple independent introductions of diverse NDM-1 strains into the ward and the resultant equipment or environmental contamination potentially contributed to their spread in the neonatal ward. Although basic control measures had been strictly implemented throughout the study period, they were fundamentally inadequate to curb CPE transmission. Only a series of measures progressively developed on the basis of a comprehensive understanding of their transmission and risk model assessment had eventually controlled the spread of these resistant organisms.

Risk factors for CRE infection and/or colonization had been widely reported in adults, but they had rarely been described in neonates. In one study, risk factors for colonization or infection with CRE in children were identified as antipseudomonal antibiotic exposure, prior surgery, and mechanical ventilation [20]. In another study, top feeding and antibiotics administration were shown to be associated with neonatal gut CRE colonization [21]. In the present study, risk factors identified for neonatal rectal colonization with CPE were not exactly the same in the two age groups: underlying conditions or diseases (acidosis and respiration failure) were only observed in the 0–3 DO group, while antibiotic exposure (cephalosporins) was only found in the 4–28 DO group. However, cesarean section and/or medical procedures (gastric lavage and/or enema) were the shared risk factors for both age groups, suggesting that inappropriate medical procedures and/or surgeries could easily facilitate the entry of these organisms into the intestines of immunologically immature neonatal patients. In addition, we found that patients from rural areas were an independent risk factor for rectal carriage, suggesting the existence and spread of NDM-1 strains in the community.

CRE infections mainly affected critically ill children with underlying illnesses who were exposed to antimicrobials [2]. In our study, all infected patients received one or more classes of antibiotics before CPE isolation, with the most frequently used classes of antibiotics being β-lactam/ β-lactamase inhibitor combinations and carbapenems. After culture susceptibility results were available, all patients received antibiotic monotherapy. Among patients with pneumonia and sepsis, 16.67% (15/90) and 6.25% (1/16) switched to active drug (ciprofloxacin), while none of the patients with KPC-2 KP infections received active antibiotic treatment. This situation could be partially explained by the limited treatment options for CPE in our setting, especially for KPC-2 KP. In one study, high mortality rate was associated with respiratory source [22]. However, in another study, all CRE infected patients had been cured or improved after monotherapy or combination therapy [11]. In our study, we found that there were differences in the outcomes between infection types. Pneumonia patients who received antibiotic monotherapy had a relatively favorable outcome, while sepsis patients who received non-active antibiotic monotherapy had a poor clinical outcome. The isolation of CRKP was the main risk factor for mortality from bloodstream infection (BSI) [23, 24]. Some diseases or serious underlying conditions such as oncologic diseases and transplant-associated immunosuppression were significantly associated with a fatal outcome among children with CRE infections [2, 22]. Non-active antibiotic treatment contributed to unfavourable outcomes in children with CRE BSI, while active or appropriate combination therapy could significantly reduce the mortality in children [2, 11, 22, 23]. We believed that the type of infection and the severity of patient’s disease or underlying condition should be considered in the selection of antibiotic therapy and that prompt initiation of appropriate combination therapy would be crucial for the survival in patients with suspicion of CPKP BSI.

In the stuy, KPC-2 KP, NDM-1 KP and IMP-4 KP could cause neonatal sepsis, and the distribution of these patients in both genders was similar. Patients with sepsis and pneumonia had relatively better baseline conditions, and none of them had documented disease risk factors associated with CRKP-related infection, such as hematologic malignancies, metabolic disease and neutropenia [24, 25]. One study showed that no specific factors were associated with BSIs in the colonized patients [26]. In our cohort, none of the rectally colonized patients developed subsequent BSI. The difficulties in the diagnosis and management of these vulnerable patients with CPE BSI predisposed them to a more serious and complicated disease courses [27]. Due to the high prevalence of CPE and the paucity of effective antibiotics in children, continued surveillance and comprehensive infection control measures were urgently needed to reduce patients’ risk of acquiring and dying from these pathogens.

The neonatal ward had experienced pandemics of diverse NDM-1 strains and finally been terminated through multifaceted intervention measures. The complete success was attributed to the following three key factors: (i) Intestinal colonization could serve as a significant reservoir for the transmission and infection of CRKP in the healthcare facilities [8, 16, 17]. Active surveillance cultures were essential in identifying CPKP and their prevention from transmission [7]. Our results also indicated that active rectal surveillance culture combined with efficient bacterial typing method could give more complete transmission information that assisted in allowing for timely targeted control measures to be taken. Previously, CPE strains were detected only based on clinical samples while the transmission caused by colonization was almost completely ignored, leading to environmental contamination and subsequent transmission. (ii) Another important component in the success of the intervention was to reduce the workload and improve compliance with control measures. Overcrowding and understaffing could lead to the breakdown of control measures, which in turn facilitated the transmission, even if patients were placed in single rooms after knowing the carriage status of multi-drug resistant (MDR) bacteria [28, 29]. One study also showed that patient/nurse ratio was associated with rectal CRKP colonization [26]. The reduction in the number of beds in each room and the increase in medical staff had substantially contributed to the control of the transmission. (iii) Risk assessment could reveal equipment- or device-associated transmission and enable precise interventions to contain the transmission [30–33], even sometimes without any bacterial evidence [32, 33]. Antibiotic use could exert selective pressure on the persistence of MDR organisms, while the restriction of the use of cephalosporins was associated with reduced acquisition with these organisms in children patients [34, 35]. Our analysis of risk factors suggested that inappropriate medical procedures and irrational use of cephalosporins were associated with silent transmission and high rectal colonization throughout the study period. Therefore, accurate evaluation of the risk factors could help identify deficiencies in currently implemented measures and then improve infection control measures.

Nosocomial spread remained the most common risk factor for CRE acquisition, but in some regions, the community acquisition might be a contributing factor in the development of CRE [1]. Our epidemiological data indicated that multiple or repeated independent importations of diverse NDM-1 producers into the pediatric and neonatal wards direct from the community through colonized and/or infected infant patients might be a driving factor for the continued transmission. Multivariate analysis also demonstrated that residence in rural area was an independent risk factor. These findings raised special concerns about the community spread of NDM-1 strains. In fact, The diffusion of *blaNDM* gene in the community in China was extremely common and their sources had been identified from animals [36, 37], fresh vegetables [38], retail meat [39] and multiple commercial farms [37, 40]. A study indicated that NDM organisms largely occurred and disseminated in the community primarily through waterborne and foodborne transmission [41]. Therefore, further studies are urgently needed to determine the sources and the transmission modes of these NDM-1 strains in the community in our region.

Many STs of CPKP had been detected in neonatal patients, such as ST11, ST14, ST15, ST16, ST20, ST37, ST54, ST70, ST76, ST105, ST147, ST278, ST1419, etc. [8, 9], Newly emerging STs continued to be reported in China [9–11]. In the study, we detected not only some documented STs but also some undocumented STs in the literature. The diversity of STs detected or reported in children suggested that horizontal dissemination of *blaNDM* gene within or across *Enterobacterales* species might be the main driving force for the pandemic of NDM-producing strains, which posed an immense challenge in designing precise control measures to disrupt their transmission.

Carbapenem-resistant hypervirulent KP (CR-hvKP) had been increasingly reported in China [42]. The majority of CR-hvKP was initially detected with *blaKPC − 2*, especially CPKP ST11, and patients with CR-hvKP infections were mainly reported in adults, while hvKP infections in neonatal patients had been rarely reported [27]. Luo et al. reported that none of their neonatal CRKP isolates carried hypervirulence genes [43]. Studies showed that most severe forms of neonatal sepsis with virulent KP had an unfavorable outcome [44]. Banerjee et al. reported that all the neonatal sepsis patients died from extensively drug-resistant and hypervirulent KP ST5235 [45]. In our study, isolates of NDM-1 producing KP ST23 (serotype K1) were confirmed to carry hypervirulence genes, but these patients were recovered after treatment. The pandemic of hyper-virulent CPKP in neonatal ward was a real challenge for neonatologists and infectious disease physicians due to the serious complications and lack of choices for treatment. Considering the epidemic spread of NDM strains in Chinese children [4] and the pandemic of hyper-virulent NDM KP, increased vigilance, ongoing surveillance and stricter appropriate control measures were essential to contain the spread of these organisms.

In conclusion, CPE would become a persistent problem in the future, largely due to the continuing risk of importation pressures from the community. Multimodal infection prevention strategies in healthcare settings should be developed and reinforced. Public awareness and hygiene campaigns to prevent the spread of CPE in the community should be greatly enhanced. Given that the NDM strains are evolving to be hyper-virulent, continued vigilance and comprehensive epidemiological studies specific for institutions or regions are urgently needed to address the future CPE challenge.

## Methods

### Study design and definition

The study was conducted in Hebei General Hospital, a 1830-bed tertiary care hospital located in Hebei, China. All patients under 1 year old who were colonized/infected by CPE in rectal screenings and clinical specimens from April 2013 to April 2018 were included in this study. The study population was stratified by age as follows: 0–3 DO, 4–28 DO, and 29 DO-1 YO for infant patients. Data regarding demographics, microbiology, epidemiological and clinical characteristics, therapy and outcome were collected via electronic medical record review. A CPE case was defined by the isolation of CPE in any biological sample obtained from the patient. Infection and colonization were classified as either community acquired or healthcare associated according to the criteria established by the U.S. Centers for Disease Control and Prevention (CDC) [46]. Only the characteristics of neonatal patients from active rectal surveillance at hospital admission and during hospitalization were compared to identify risk factors for CPE acquisition. Patients with rectal CPE colonization were categorized as the case group, while control patients were patients with CSE isolated from rectal cultures.

### Microbial identification and antibiotic susceptibility testing

Different plates or media were routinely selected according to different specimens, and the inoculated media were incubated aerobically at 35 °C for at least 48 h. Sputum sample was streaked onto blood agar plate, chocolate agar plate and eosin methylene blue (EMB) agar plate. Urine sample or wound swab (pus swab) was inoculated onto blood agar plate and EMB agar plate. Blood samples or body fluids were inoculated directly onto blood culture bottles, and these inoculated bottles were incubated and inspected for 5 consecutive days. When cultured bottles showed signs of microbial growth, sub-cultures were immediately inoculated onto blood agar plates and EMB agar plates. Screening samples (stool, rectal/perirectal swabs and amniotic fluid specimens from vagina after rupture of membranes) and environmental samples were streaked onto blood agar, EMB agar with or without meropenem (1 mg/L) and incubated aerobically at 35 °C for at least 48 h. The suspected colonies grown on the selective medium after incubation were subcultured onto Mueller–Hinton agar on which 10 μg meropenem disk and 10 μg imipenem disk were placed which allowed the establishment of optimal zone diameters for the screening of CRE. After incubation for 24 h at 35 °C, a reduced zone of inhibition of ≤ 22 mm around the meropenem and/or imipenem disc was considered a potential CRE. Bacterial identification and susceptibility testing were routinely performed using the Vitek 2 automated system (bioMe´rieux) and the AST-GN09 card following the manufacturer’s instructions, and the results were interpreted according to the criteria of the latest Clinical Laboratory Standards Institute (CLSI) document. If necessary, MALDI-TOF MS was used for species verification. Meropenem and imipenem susceptibilities were also carried out by the Kirby-Bauer disk diffusion method on Mueller–Hinton agar for confirmation if a discrepancy in susceptibilities between carbapenems by Vitek 2 occurred. Carbapenem resistance was defined as nonsusceptibility to imipenem or/and meropenem or carbapenemase genes positive. *E. coli* ATCC 25922 was used as a quality control.

### Detection of carbapenemase genes, virulence-associated genes and bacterial typing

The carbapenemase genes of isolates with reduced susceptibility to carbapenems were tested by PCR, including class A (*blaKPC*, *blaNMC*, *blaGES*, *blaIMI* and *blaSME*), class B (*blaVIM*, *blaIMP*, *blaNDM*, *blaSIM*, *blaSPM* and *blaGIM*) and class D (*blaOXA-48*) [47, 48]. Virulence-associated genes (*magA*, *kfu*, *allS*, *iutA*, *rmpA*, *rmpA2*, *iucA*, *aerobactin* and *iroN*) were identified as described previously [49, 50]. Bacterial typing was carried out by multilocus sequence typing (MLST) using primers listed in the online databases (https://bigsdb.pasteur.fr/klebsiella/klebsiella.html for KP and https://bigsdb.pasteur.fr/ecoli/primers_used.html for EC). Amplicons were sequenced and compared with the reported sequences from GenBank by Blast.

### Intervention measures

No special guidelines regarding CRE prevention in China existed due to the lack of evidence-based epidemiology. Basic infection control measures for CRE employed were based primarily on experience with other MDR organisms, which included hand hygiene, contact precautions and/or isolation precautions, healthcare staff education, environmental cleaning and disinfection, aseptic procedures, and antimicrobial stewardship policies.

When recognizing outbreaks, an infection control task force was immediately formed, and multidisciplinary meetings were frequently conducted to identify additional effective control measures based on clinical observations, epidemiological surveys and molecular evidence. Monthly environmental surveillance cultures, including air sedimentation cultures, hand swab cultures, and environmental swab cultures which involved high-use medical devices, frequently touched common areas, and areas surrounding the patients, were routinely performed in affected wards, especially ICU, maternity, neonatal, and pediatric wards. Amniotic fluid and rectal swabs or stool from pregnant women were also collected during the study period. These screenings were most likely based on the following significant assumptions: (i) Prior to 2015, the neonatal ward and ICU were located on the same floor, and both wards experienced the epidemic of KPC-2 KP strains; (ii) Pregnant women with suspected intrauterine infections might carry these bacteria, which could be transmitted to the newborns during delivery; (iii) Junior medical staff generally rotated on a regular basis between neonatal and pediatric wards, and their movement might be a contributory factor.

To confirm whether infant patients carried CPE in the intestine, we initiated a first prospective rectal screening for the presence of CPE on their admission and during hospitalization during the period from 20^th^ of September 2015 to 30^th^ of April 2017. To observe the efficacy of newly instituted prevention measures on the neonatal colonization, a second prospective rectal surveillance for CPE colonization was performed from September 15, 2017 to April 30, 2018.

### Statistical analysis

Data were presented as the mean ± SD, number, percentage, or frequency. Student’s t test or Mann–Whitney U test and Chi-squared or Fisher’s exact test were used to compare qualitative and categorical variables, respectively. All variables with a *P* value ≤ 0.1 in the univariate analysis or that were known risk factors for CRE were included in a backward selection multivariable logistic regression model to identify risk factors independently associated with rectal colonization of CPE. Odds ratios (OR) with 95% confidence intervals (CI) were calculated. All tests were two-tailed, with a *P* value of < 0.05 considered statistically significant. All analyses were performed using SPSS software.

## Data Availability

The datasets used and analysed during the current study are available from the corresponding author on reasonable request.

## Abbreviations

CSE: Carbapenem-susceptible *Enterobacterales*
CRE: Carbapenem-resistant *Enterobacterales*
CPE: Carbapenemase-producing *Enterobacterales*
CRO: Carbapenem-resistant organism
KP: *Klebsiella pneumoniae*
EC: *Escherichia coli*
CRKP: Carbapenem-resistant *Klebsiella pneumoniae*
CPKP: Carbapenemase-producing *Klebsiella pneumoniae*
CPEC: Carbapenemase-producing *Escherichia coli*
CR-hvKP: Carbapenem-resistant hypervirulent KP
MDR: Multi-drug resistant
BSI: Bloodstream infection
ST: Sequence type
DO: Days old
YO: Year old
